# Achieving energy justice in Malawi: from key challenges to policy recommendations

**DOI:** 10.1007/s10584-022-03314-1

**Published:** 2022-02-12

**Authors:** Darren McCauley, Rebecca Grant, Evance Mwathunga

**Affiliations:** 1 Management of International Social Challenges, Erasmus School of Social and Behavioural Sciences, Department of Public Administration and Sociology, Erasmus University Rotterdam, Rotterdam, Netherlands; 2 School of Geosciences, University of Edinburgh, Edinburgh, Scotland; 3 Department of Geography & Earth Sciences, University of Malawi, Zomba, Malawi

**Keywords:** Energy justice, Environmental justice, Gender, Social inequalities, Energy policy, Energy transitions

## Abstract

Addressing energy provision and access in Sub-Saharan Africa is a key global challenge. Drawing on interviews with key stakeholders, this paper applies an energy justice framework in overviewing energy realities and policies in Malawi, where electricity access remains among the lowest in Sub-Saharan Africa. The use of woodfuel remains high for meeting cooking, heating, and lighting needs leading to indoor air pollution, with serious health consequences, and widespread deforestation. Responses to these dual challenges, a lack of electricity access and ongoing woodfuel use, must be rooted in notions of equity, fairness, and justice. Application of energy justice theorising provides insights into how policy stakeholders are responding to complex and interconnected issues of energy generation and access in low-income settings. Overall, a just response to these energy challenges is possible, but only if it is built on local inclusive governance with fairer and effective systems of investment.

## Introduction

**Fig. 1:**
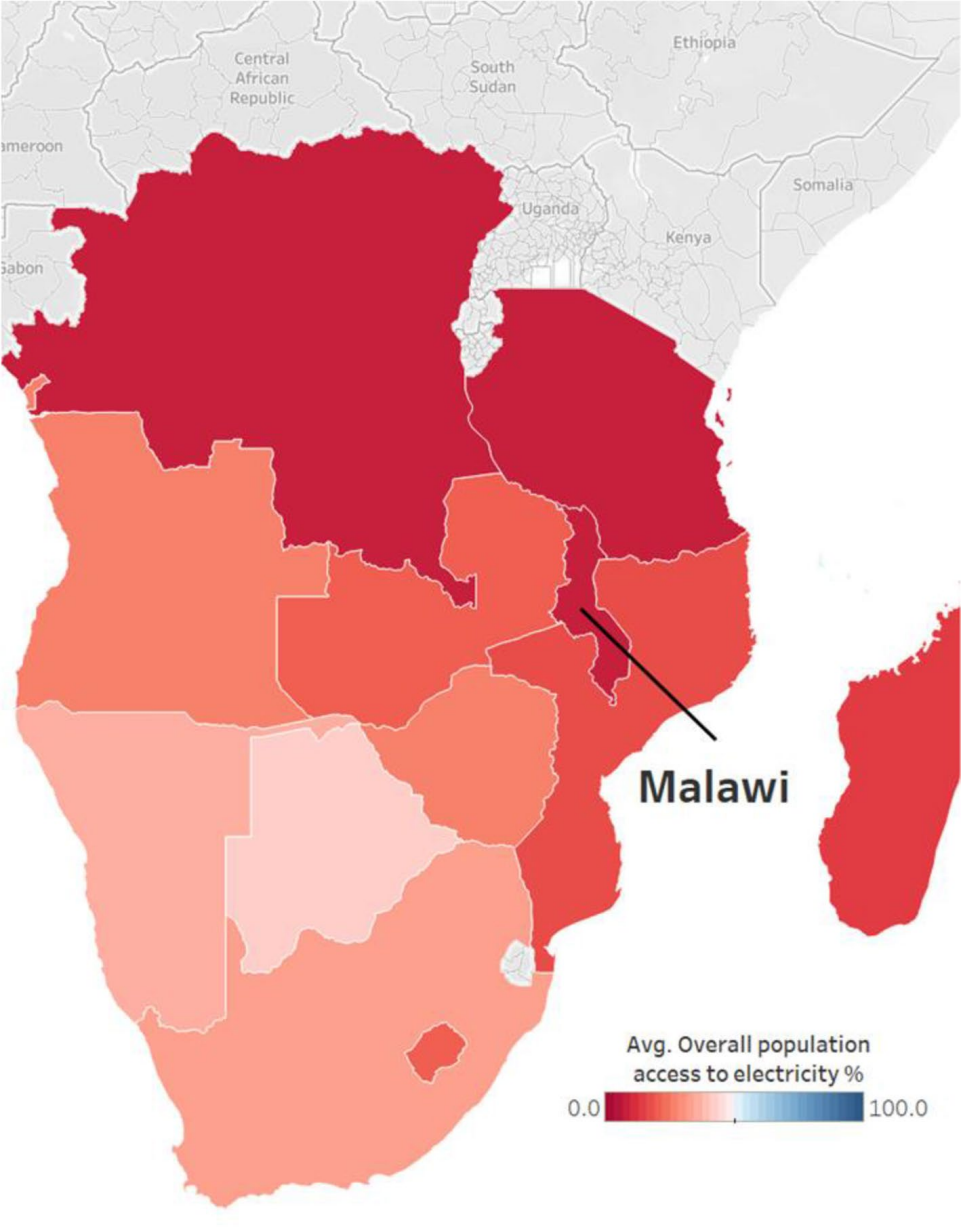
Average electricity access (%) in Malawi in the context of Southern Africa, 2010–2020: This map shows United Nations Development Programme’s (UNDP) % population with access to electricity for 2010–2020 ((UNSTATS 2021). Nations in dark blue have higher levels of electricity access (the highest is Botswana at 41%), and those in darker shades of red have the lowest access. Malawi has the lowest average population access to electricity in Southern Africa (7.2%), followed by DR Congo (7.8%) and Tanzania (8.8%)

Despite recent improvements to electricity access in Malawi, access to electricity remains at just 13.4% of the population (IEA 2020) lower than the Sub-Saharan African regional average of 47.9% (ibid). Figure 1 below sets out the average electricity access (%) in Malawi in the context of Southern Africa, 2010–2020. It shows Malawi as having the lowest average population access to electricity (7.2%) in Southern Africa, followed by DR Congo and Tanzania (7.8% and 8.8% population access, respectively). The impacts of both COVID-19 and population growth threaten to increase absolute numbers of those without access to electricity in Malawi (WB 2021). Both, a lack of sufficient and reliable energy supply and limits to grid extension, hinder rollout of electrification and expansion of access (Arndt et al. 2014; Kaunda 2013).

Systems of hydropower dominate electricity generation, with 372 MW installed hydroelectricity capacity out of a national total of 532 MW (USAID 2019). However, vulnerability to climate changes is increased by an overreliance on hydropower for energy provision; a drought in Malawi led to a nationwide blackout in December 2017, and modelling, conducted by the IPCC and reported in the 2019 African Energy Outlook (IEA 2019), predicts decreasing capacity of hydropower with climate changes. Malawi’s Ministry of Natural Resources, Energy, and Mining announced in October 2018 that it must import electricity from Zambia in light of the significant threat of blackouts (Samarakoon 2020). The Chinese-backed Kam’mwamba Coal Power Station offers to increase installed capacity in the country by 300 MW, almost doubling the existing national electricity capacity. It is to be based outside the city of Blantyre in the south of Malawi, with the coal delivered from Mozambique. This contrasts with the stated aim of international organisations such as the World Bank to enable more renewable energy deployment in Malawi, and global mandates such as those in COP26 which are targeting finance for renewable energies in low-income countries (WB 2021). As of February 2021, the ministry has not secured the $667 million needed from China’s Export and Import Bank (Exim). At this critical energy crossroads, we assess how best to ensure clean future energy provision, increase electricity, and clean cooking access while protecting the environment in a fair and equitable manner.

**Fig. 2:**
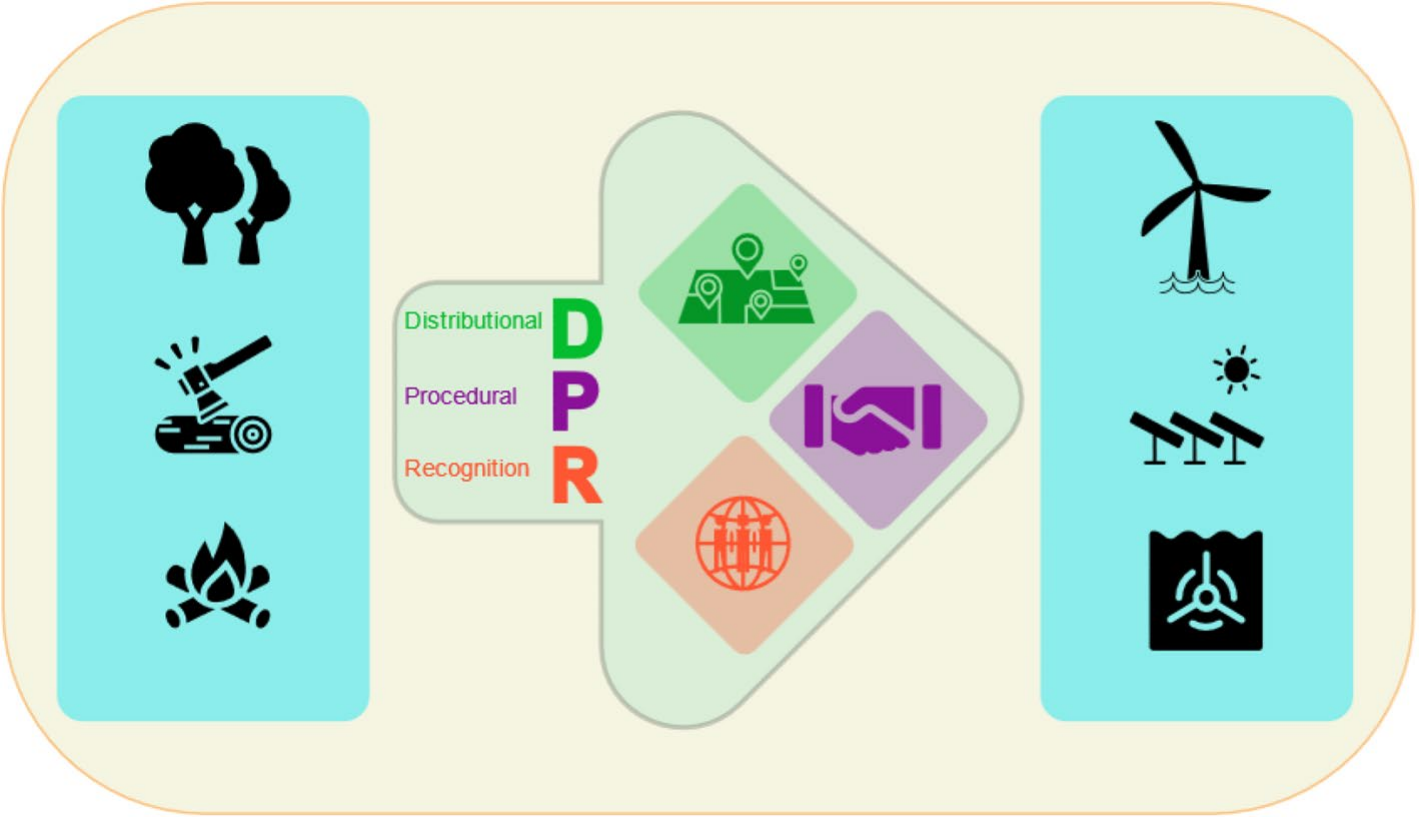
An energy justice framework for assessing the energy transition in Malawi. This infographic represents the energy transition (pale green arrow) in Malawi from the use of wood-based energy sources (showed by the blue panel on the left: trees and forests, wood-felling, and wood burning) to renewable energy technologies (showed by the blue panel on the right; wind turbine, solar panels, and tidal turbine). The green arrow represents the exploration of this transition through the lens of distributional justice, procedural justice, and recognition justice. Distributional Justice is represented by the green map icon, which refers to the geographical distribution of social inequalities. Procedural Justice, represented by the purple handshake icon, refers to the exclusion of those most affected by injustice from decision-making processes. Recognition Justice is represented by the red globe icon and refers to those groups who are most affected

Energy justice offers a way to capture these concerns. There are several approaches to understanding and applying energy justice, including life cycle approaches, three tenets, energy justice principles, and whole systems approach (Jenkins et al. 2020). We utilise the Energy Justice Framework to assess questions of fairness and equity of both energy production and consumption, in rural and urban areas, from the household and village communities to the national and international levels. This approach assesses distributional (where social inequalities emerge), recognition (who is affected), and procedural (when those affected are overlooked in decision-making) injustices (Feenstra and Özerol 2021; Thomas et al. 2020; Velasco-Herrejon and Bauwens 2020; Wood and Roelich 2020). This paper draws on the three-tenet approach to energy justice to assessing the energy transition in Malawi (see Fig. 2), examining opportunities to expand theorising on energy justice to settings of pre- and ongoing electrification in Malawi.

Energy justice, which emerged from the environmental justice movement and concerns over the unequal spatial and temporal impacts of energy system siting in the USA, refers to a diverse body of academic contributions seeking to identify, locate, and examine the emergence of injustices across energy supply chains (Pellegrini-Masini et al. 2020). Despite this diversity, Pellegrini-Masini et al. (2020) argue that definitions of energy justice are united by a common vision for equality (formal and substantive) coupled with a concern over historic and ongoing inequalities relating to impacts and engagement in energy systems.

One of the most commonly applied frameworks of energy justice across academic case studies is the three tenets of energy justice which we draw on in this paper (Lacey-Barnacle et al. 2020). These include distributive justice, concerned with the unequal distribution of benefits and risks emerging across energy supply chains, procedural justice, and the pursuit of fair, inclusive, and accessible involvement in procedures governing systems of construction, distribution, use and impacts, and recognition justice, and the full inclusion and recognition of all affected stakeholders (Jenkins et al. 2016). Other frameworks understand energy justice according to 10 key principles, which cover availability, affordability, intergenerational and intra-generational equity, due process, accountability, transparency, and intersectionality (Sovacool et al. 2017). This diversity and dissonance in definitions, however, has arguably come at the cost of application of energy justice principles to policy (Pellegrini-Masini et al. 2020).

Case studies observed through the lens of energy justice originally focused primarily on systems of large-scale on-grid electricity generation in the Global North (Jenkins et al. 2020). However, case studies are increasingly examining injustices in lower income, off-grid, and pre-electrification case studies globally, including in sub- Saharan Africa (Boamah 2020; Boamah et al. 2021; Todd and McCauley 2021a, b). Alongside critiques of a western-centric focus in case studies energy justice (see Lacey-Barnacle et al. 2020), authors are increasingly looking beyond anthropocentric experiences and conceptualisations of injustice to incorporate the intersecting, and unequal, impacts of energy systems on human wellbeing and ecosystem health (Grant et al. 2021).

Our aim in this paper is to explore how public, private, and third sector organisations based throughout Malawi (see a list of organisations interviewed in Appendix) identify, describe, and explain the energy challenges Malawi faces, through an energy justice lens. Our research data draws on qualitative interviews undertaken in Malawi in March and June 2019. The authors utilised the energy justice framework of distributional, recognition, and procedural justice to probe and analyse interview data on the energy policy and financing landscape in Malawi. We sought to understand, from those working in the policy sphere, the challenges impacting on electrification and energy generation in Malawi and the solutions proposed to address these. The paper is based on thirty interviews with representatives from international organisations, national government, non-governmental organisations (NGOs), and businesses. We assess the discourse produced by each policy stakeholder interviewed, coding the resulting data based upon the energy justice framework. A literature review on energy challenges in Malawi and the energy justice framework is set out below to extrapolate key existing themes relevant to our energy justice research in Malawi.

## Literature review

We review existing literature on the main energy challenges facing Malawians and policymakers before moving on to explain the energy justice framework more fully in relation to current literature. We performed a literature search on energy challenges in Malawi and then energy justice and policy through google scholar, focusing on Scopus ranked journals. For a more comprehensive and systematic review of literature on energy justice more broadly, please see Jenkins et al. (2020). The literature reviewed here is used to inform our research and policy recommendations, which are outlined in the proceeding sections.

### Energy challenges in Malawi

Concerns relating to low levels of electrification and clean cooking access continue to dominate the research landscape in Malawi (Arndt et al. 2014; Aung et al. 2021; Barry et al. 2011; Gamula et al. 2013; Kaunda 2013; Onyeji et al. 2012; Tchereni et al. 2013). Energy poverty in Malawi has been defined as an ‘endemic’ widespread ‘state of deprivation where households can barely meet the most the minimum energy requirements for basic needs’ by Tchereni et al. (2013), whose research focuses on the extent of energy access issues across households in both urban and rural Malawi. Widespread energy poverty has also been confirmed by census data from the Government of Malawi (Malawi 2017, 2018). Within this context, more recent work by Aung et al. (2021) has highlighted the challenges of what is termed as the ‘ultra-poor’, which are households in Malawi that are primarily rural and are beyond both national grid and off grid energy generation networks. Aung et al. (2021) show, in contrast to previous research, that substantial variation exists between better-off households and ultra-poor households.

This leads us to a second challenge, relating to the establishment of bottom-up community or village-based energy schemes. This strand of research in Malawi has presented examples of community-based projects in rural areas, also highlighting challenges which projects face with respect to funding, objective setting, and competing priorities (Adkins et al. 2010; Jagger et al. 2017; O'Shaughnessy et al. 2014; Owen et al. 2013; Toth et al. 2019; Trotter 2016). Access to electricity and clean cooking remains a core theme through which to understand energy challenges in the country. These projects also encourage wider connected non-energy changes such as sustainable practices or new skills development, with literature examining co-benefits or unexpected challenges which emerge in projects with multiple objectives (e.g. relating to long-term financial sustainability and the establishment of key objectives). Better organised and systematically funded rural communities are positioned as a solution for inadequate access to electricity and heating. An example is the Millennium Villages project which started in 2004 and represented over 80 villages across ten sub-Saharan countries, including Malawi. It has developed whole-system changes that have provided alternative forms of self-sustaining economic growth from technological development to new farming techniques (Adkins et al. 2010). Such research has built on the power of community participation and involvement.

Additional understudied challenges in the literature on Malawi highlight interconnected research themes relating to limits to expansion of individual- and community-level access to electricity relating to finance and regulatory environments enabling investment. Chirambo (2016) shows, for example, that a national-level combination of price-guarantee schemes, cross-subsidies, and environmental taxes would cause higher levels of international energy sector investment. There has also been some recent critical reflection on the effectiveness of international renewable investment in Malawi in relation to systems of off-grid solar (Samarakoon 2020).

A fourth challenge emerges in relation to how best to achieve better outcomes in non-energy services such as health, food, or the environment through the development of related energy infrastructure. Suhlrie et al. (2018) demonstrate how intermittent and ineffective electricity generation affects health facilities in Malawi, especially night-time care services. They call for a health-driven wave of investment in energy infrastructure. Schuenemann et al. (2018) show how a sustainable approach to the biomass energy sector in Malawi could lead to higher levels of food security. We assess these and other challenges through the energy justice three tenet approach.

### Addressing key policy challenges through the energy justice three-tenet approach

Energy justice is defined as the fair and equitable application of rights across systems of energy provision, access, and associated environmental services. It encourages researchers to broaden analyses beyond technical or engineering-based solutions to energy system problems (Jenkins et al. 2020; McCauley et al. 2013; Welton & Eisen 2019). It is also a valuable tool in understanding how inequalities such as poverty, gender, and social class interact within the context of energy policymaking, systems, and infrastructures (Heffron et al. 2018; McCauley et al. 2019; Sovacool et al. 2020). Too few studies attempt to reflect on these interconnections in countries like Malawi (Lacey-Barnacle et al. 2020). We argue that our contribution adds a much-needed response.

We apply the energy justice framework to understand how policy stakeholders in Malawi view the distributional, recognition, and procedural energy injustices of the four key energy challenges we have identified in the literature, and others yet to be identified. In this way, it can assess energy policy challenges and help to offer practical recommendations (Heffron et al. 2018; Jenkins et al. 2017). Marlin-Tackie et al. (2020) argue, for example, that their energy justice analysis points towards the urgent need to enhance local government to better deal with fracking. New policy mechanisms have been identified in relation to local decision-making in Bangladesh through the energy justice framework (Moniruzzaman & Day, 2020). Kruger and McCauley (2020) put forward recommendations for financial compensation in hydropower policy Democratic Republic of Congo. The focus of such research is to explore the expert opinions of key policy stakeholders at all levels.

Energy justice in policy literature sets out a ‘trade-off’-based understanding that is well established in existing policy analysis literature (Gunningham 2013; Kosai and Tan 2017; Oliver and Sovacool 2017). The overarching aim is to achieve a balance between competing interconnected policy objectives (Shah et al. 2021; Song et al. 2017; Sprajc et al. 2019). Analytical tools such as the energy policy trilemma have been applied in both quantitative research (Heffron et al. 2018) to capture trade-offs better. However, it is yet to be applied to qualitative field-based research. Energy policy trilemma research on justice indicates that there are three critical interconnected objectives concerning (i) increasing secure energy provision in energy generation systems, (ii) expanding electricity access and access to clean cooking solutions to reduce poverty, and (iii) commitment to promoting environmental stewardship and sustainability (Heffron et al. 2018; McCauley 2018). Existing literature argues that a balance between all three priorities is ideal for achieving energy justice. In the conclusion, we reflect on where policy recommendations are most urgently needed in Malawi.

## Methods

Our research data is based on interviews undertaken in Malawi in 2019, with questions and coding guided by the Energy Justice Framework. Our methodology is rooted in qualitative research approaches (Bell et al. 2012), guided by a constructivist philosophical paradigm.

Constructivists argue that the focus should be on how the world is shaped (or constructed) by the way people communicate about it via speech, visual cues, and/or text (Houston 2013; Sheftel & Zembrzycki 2013). These visions and experiences, or storylines, are constructed by policymakers using a variety of discursive categories to make sense of phenomena, e.g. energy challenges and solutions (Moore et al. 2017). Analysing storylines produced in verbal interviews with policymakers allows us to better understand how key stakeholders construct their understanding of justice in relation to agency and power.

We aimed to uncover the justice dimensions of the viewpoints held by key policy stakeholders based throughout Malawi. We restricted the research timeframe to 2019 when the Malawian government was (and still is at the time of writing) considering the Chinese-funded Kam’mwamba Coal Power Station. We developed our overall research question from the literature review on energy justice, as outlined above. Our aim was to understand how policymakers understand the justice dimensions of the key energy challenges in Malawi. The energy justice framework argues that energy justice can be understood as relating to distributional, recognition, and procedural injustices (McCauley et al. 2019). Our interviews investigated who we should recognise as the most affected by energy scarcity and the key actors driving the current energy policy agenda. We also asked interviewees to assess how communities were engaged in energy decision-making to better understand energy injustices.

We conducted 30 interviews with representatives from three key policy stakeholder groups, i.e. government (8), NGOs (12), and businesses (10). We sought gender balance in our interviewees with a final breakdown of 14 female and 16 male interviewees. A full list of organisations of all types is in Appendix 1. All interviews took place in Blantyre and Mulanje (home to the off-grid first Independent Power Producer in Malawi) in the south, and the capital Lilongwe in central Malawi. Out of Malawi’s total population of 17.5 million, the southern region has the highest population, with 44 percent of the total population. The Central Region is the second most populous, according to national census data in 2018, with 43 percent of the population, while the northern region population makes up 13 percent of the total population.

We numbered all thirty interviews, randomised their order, and then presented them in text as (#1), (#2), etc. with the following according to our three analytical categories: government,^1^ NGOs,^2^ and business.^3^ Our aim was to assess the discourse produced by each stakeholder interviewed to code for energy injustices, recognising that the language (and meaning) of ‘justice’ is not shared by all and focusing instead on a language of risks, benefits, and opportunities to capture injustice. This allows us to then select key quotations from our different interviewees. These are then the basis for our qualitative data, as explored in our results below. The three dimensions of distribution, recognition, and procedural injustices were used as key starting points for identifying critical citations. The interviews were semi-structured in nature but were in keeping with these three themes and categories.

We also added a fourth category that focused on questions around energy consumption and production to determine the nuances within each of these processes. This was to make sure we covered both issues covering access to electricity and clean cooking solutions, and provision issues raised by our interviewees. As outlined in our literature review above, electricity access or consumption is only one challenge. A fuller spectrum of energy challenges is then addressed in our analysis, as detailed in the results below. Literature, reports, and selected newspapers were consulted to develop a triangulated robust account of stakeholder interpretations. We did not seek to code these documents, as the focus was on the interviews. They allowed us to corroborate some statements from interviewees and key events.

We completed a full ethical and risk assessment and an additional process set out by the Malawian National Committee on Research in the Social Sciences and Humanities. Research aims were made clear through a statement of intent, and participants were required to give their consent. Participants were asked to give their consent for audio data to be recorded where possible, for data use in analysis and data presentation. Data was stored in a fully identifiable format on a computer. Participants were given the opportunity to ask questions regarding the project and to withdraw at any point. All ethical and risk assessment forms are available upon request.

## Analysis

We present below our assessment of the interview data based on our three primary qualitative codes, i.e. distributional, recognition and procedural injustices. Insights into the processes of energy production and consumption were coded separately but linked to three primary qualitative codes and enabled us to triangulate insights. We integrate results from the fourth coding category of energy production and consumption into the three justice dimensions and helped us to triangulate insights provided by our interviewees. As outlined above in the methods, the number denotes the interview source in brackets, such as (#12). We reflect throughout on how these results connect to existing literature, before moving into specific policy recommendations in the last section of the paper.

### Distributional justice

Our interviewees pointed to the unequal distribution of benefits from existing electricity generation and transmission. The average connectivity of the Malawian population to the national grid is at 11% (Gov 2017; Malawi 2018). While energy poverty is widespread, access rates differ between urban and rural regions, and between regions. Access to the national grid in cities is higher than in rural areas, a well-reported finding from research elsewhere (Banerjee 2018; Caniglia et al. 2017; Gossling 2016; Kelly-Reif and Wing 2016; Perez and Egan 2016). For Malawi, one of the key issues identified was a lack of financial capacity to afford electricity connection and services (#3). The interviews revealed a persistent focus on urban communities as proximate to existing national grid networks, and of concerns relating to ability to pay in rural regions limiting expansion (#3, #5, #8). Lack of on-grid electrification, coupled with descriptions of spatially ‘random’ positioning of off-grid electricity schemes in rural regions, contributed to an environment where businesses were described as moving to urban areas to optimise trade (#10, #15). An unequal distribution of benefits is clear not only along rural/urban spatial descriptors but also at a regional level.

The northern parts of Malawi have least access to electricity compared to the central and southern regions where important cities such as Lilongwe and Blantyre are located (#16). Electricity lines in the north of Malawi were described as being of lower voltage (#21), with those in rural locations in northern Malawi described as the most disadvantaged in the country with respect to quality of connection. However, interviewees also expressed significant concern for the south of the country given the severe deforestation ongoing in the region (#17, #18). The clearance of land for agricultural purposes, observed in the surrounding areas of Mulanje, contributed to scenarios where residents were described as having to walk up to 10 km (#17) to collect firewood for cooking, heating, and, in some households, lighting. This was contrasted with those in northern regions, where deforestation was less extensive and demand for fuelwood lower in Mzuzu (in part due to lower population density) (#17).

Distributional injustices also relate to proximity to renewable energy resources such as hydropower. Interviewees in Mulanje highlighted that those living close to mini-grid hydropower benefit through reliable access to electricity and job creation tied to development (e.g. in Mulanje Electricity Generation Agency) when compared to other living further away from facilities of power generation (e.g. those upstream or downstream) (#17).

Interviews also attested to a strong link between distributional variation in electricity access and the inequalities experienced between different consumer groups. Where resources to expand generation capacity and extend national grid infrastructure are limited, industrial development was frequently siloed to cities such as Lilongwe. This was also reflected in approaches to energy planning which prioritised regions conceptualised as having the highest growth potential: ‘the south [is] more developed and more industrial…the growth rate is different and the center is growing the fastest…while the north is much smaller’ (#2). Electrification planning is focused on expanding access in an increasingly industrialised central and southern Malawi (close to major cities). Steel processing, agro-processing of sugar, cotton and mangoes, and mining were identified in the south as the most power-intensive industries (#2). An interviewee (#5) judged that World Bank-funded solar projects in the North were a ‘vanity project’ that national government could not afford: ‘Industry in the South needs to encourage large scale/bulk buy-in (of electricity)’ to make national energy planning more effective (#8).

Beyond industrial demand, there are concerns regarding the economic sustainability of electricity expansion in rural areas given lack of ability to pay. Many households opt instead for charcoal use to meet heating, cooking, and lighting needs (#4, #23). Markets for charcoal remain dominant especially in peri-urban and rural regions including Mulanje, with regulations seeking to limit the sale of charcoal having limited efficacy in reducing unsustainable deforestation for fuelwood (#18). The use of mini-grid renewable energy generation for expanding access to electricity in rural regions was described as incompatible given low purchasing power and the lower levels of investment in northern regions of Malawi. This, in turn, entrenches the ongoing high use of fuelwood and charcoal (#18). Promises tied to the development of large-scale solar projects, such as JCM solar, in the Northern regions of Malawi are already linked to a set of distributional injustices linked to the export of electricity produced locally to larger cities (#23, #21) (see also case study on JCM solar (Horst et al. 2021)).

Distributional inequalities arise also according to income. Higher income populations live in urban areas, with greater access to electricity provided through on-grid connection (#4). In contrast to global dialogues which consider electricity as a right, discourses from some interviewees indicated that, for lower income settings in rural areas, electricity was instead conceptualised as a privilege (#6). Widespread poverty and income inequality correlated with rurality defines electrification planning of ESCOM, the primary energy company in Malawi; a lack of economic incentives and low ability to pay were described as explanatory factors behind lack of expansion to rural regions by ESCOM (#8, #4). The ‘long-term dearth of financial capacity’ among lower-income groups is leading to a lock-in of social class-based distributional and temporal disparities, with present low ability defining future potential for connection (#21). Other interviewees argued, in contrast, that most socio-economic categories can afford a basic level of electricity. This dialogue of deservedness which defines electrification planning is underplayed, given existing challenges of generation meeting limited present demand (#9).

In Malawi, distributive injustices arise in energy system planning, in construction and in rollout of energy generation and transmission. A focus on urban, grid connected, and high economic growth potential areas both entrenches and is symbolic of a planning rationale dominated by ability to pay in resource-constrained settings. The spatially uneven distribution of planned and ongoing construction of energy systems, such as hydroelectricity schemes on the Shireriver in the south and descriptions of random siting on mini-grids, magnifies both inequalities in access to electricity (and quality of supply) and injustice tied to ongoing use of woodfuels. Distributive injustices linked to trade-offs, for example, in prioritising industrial centres at the expense of expanding the natural grid in northern and southern Malawi, are emerging already; deforestation threatens livelihoods and hampers the efficiency of existing systems of energy generation, and poor-quality electricity access further alienates geographically disparate populations in the north. Unlike mapping energy injustice in grid-developed settings in the Global North, neglecting to incorporate the physical value and symbolism of grid-connection (see Boamah et al. 2021) in Malawi will risk entrenching existing distributive injustices.

As a final comment on global distributive injustice, both the negative impacts of climate change, and impacts of decarbonisation, are unequally distributed globally (Lehmann and Tittor 2021). Our research points to adverse impacts of climate change, through increasing periods of drought and flash flooding, on livelihoods and energy generation in Malawi. Simultaneously, at an energy crossroads, Malawi risks a high-carbon lock-in under current energy scenario planning (see Alova et al. (2021) for wider discussion on this) or stagnating electricity generation with over-reliance on hydropower (see also Alova et al. 2021). Attention must be paid to the ‘triple inequalities’ of decarbonisation (see above), and the need for diverse energy mixes to meet multiple (and evolving) energy needs in systems of low and uneven electricity access (Kumar et al. 2021).

### Recognition justice

Identifying recognition injustices is key to addressing overall energy injustice in Malawi. The interviews showed two key stakeholders in Malawi who are too often overlooked by the lack of electrification which extend discourses of energy justice beyond the Anthropocene (Pellegrini-Masini et al. 2020). These include the environment and women.

First, stakeholders identified the environment as a prominent, underrepresented, and often homogenised stakeholder in discussions on energy planning (#3, #12, #16, #17). Our interviews juxtapose the contravening intrinsic and monetary values of environment with devastating effects of lack of inclusion in energy policy (as explored in the discussion). The environment in everyday translation was most frequently related to trees; interviewees simultaneously described trees as ‘backbone to African societies’ and in Malawi the ‘environment equals trees’ (#12). A custodian responsibility for their management argued that trees cut through ‘every section of society’ (#13). Contrary to assumption, one interviewee commented, ‘people do make a connection between cutting trees and climate change’ (#15). We caution, however, against extrapolating the conflation of trees with environment with wider populations (risking the romanticisation of certain ways of connecting to spaces).

However, dialogues concerning the monetary value of trees also dominate especially where poverty is endemic. Interviewees commented that ‘when people are in survival mode, you don’t think about the environment’ with firewood free (#2), with others also noting that, in conditions of extreme poverty, deforestation for fuelwood is essential even where there is concern for the impact of this behaviour (#2, #7, #15, #16, #18). Ongoing use of fuelwood for energy has resulted in rapid and large-scale deforestation in the country’s more populated central and southern regions (#2, #4,#17, #18). Communities turn to producing and selling charcoal as a source of generating income, even though such practices are illegal (#11, #17, #18). Further practices that were identified as promoting deforestation are brick manufacturing.

Stakeholders’ perceptions indicated a disregard for sustainable forestry management at government level; one stakeholder noted that the ‘Malawian government knows what climate change is’ (#16); however, ‘between awareness and action there is a huge disconnect’ (#3). The country’s most prominent source of energy generation, hydropower, plays a role in deforestation. Deforestation upstream limits the capacity of hydropower systems, with demand for timber increasingly needed in planning and construction, in addition to aforementioned agricultural and fuelwood uses (#9, #14, #15, #17). They identified population growth as another variable, exerting pressure on forests (#16). Large-scale deforestation increases vulnerability to climate shocks through increasing soil instability and vulnerability to shocks through periods of extreme rainfall and flooding (#16, #17). Arguably a focus on hydropower, coupled with limited expansion of electricity access, has led to an environment where deforestation is widespread and is indicative of lack of full inclusion of the environment, as a complex and interconnected ecosystem, in long-term electrification planning. Existing research suggests that this could have a profound long-term impact in Malawi, as past environmental policy failures affect a society's ability to view new futures (Blom-Hansen 1997; Béland 2009; Feindt 2010; Jacobsson and Lauber 2006; Leach 2008).

Second, the interviewees acknowledged that women are experiencing the brunt of the adverse effects of energy scarcity in Malawi (#1, #3, #4, #5, #11, #12, #15, #17, #18, #23). This finding complements existing research (Castan Broto et al. 2018; Damgaard et al. 2017; Islar et al. 2017; Mostert and van Niekerk 2018; Munro et al. 2017) with new empirical insight into Malawi; ‘in Malawi, those who are most affected are the ladies, the females’ (#18). A lack of access to electricity, and ongoing use of woodfuel for heating and cooking, adversely impacts of women with responsibilities over household tasks including cooking and collecting resources for this (Halff et al. 2015; Jagger et al. 2017; Munro et al. 2017; Tang and Liao 2014). Conversely, household financial management was described as a male responsibility leading to a juxtaposition where ‘Wood collection is a woman’s task. Cooking is a woman’s task. Yet financing is a man’s task, so saving wood is not important to men’ (#11).

With increasing deforestation, those responsible for collecting firewood travel greater distances to source materials: ‘Before the 2000s, women would walk shorter distances to collect firewood. Because the wood was cut, they now travel long distances. These days it is not easy. You can spend 1–2 h where you used to spend 10 min’ (#18). Collecting woodfuel increases vulnerability to risks of sexual assault and rape, especially when carried out in darkness or alone, with interviewees describing this as common in both nationally managed and privately managed areas (#4, #11, #17, #18, #23). When women are caught stealing the firewood by the guards and rangers, these abuse their power and demand ‘for terrible things from women […] sexual favours’ (#23). This was questioned by government officials, with some attributing violence against women instead to those managing tea estates (#18, #23). When probed as to solutions, interviewees argued that alleviating gender-based violence relied on encouragement of women not to harvest firewood alone (#18). Contentions over the allocation of responsibility for safeguarding against violence linked to deforestation highlight complexities in understanding fully the means through which energy injustices emergence (Toth et al., 2019).

Jenkins et al. (2016) argue that recognition injustices manifest not only as a lack of recognition or inclusion, but also in the processes of misrecognition. The environment is misrecognised (and therefore underrepresented) in two critical ways: in its conflation with trees and in its conceptualisation as a homogeneous category and stakeholder. This misrecognition in energy policy could lead to minimising or misrepresenting both the ecosystem and human health risks, and co-benefits, linked to different electrification strategies. As a wider critique of energy justice, recognising the ‘environment’ as a stakeholder in energy systems is lacking (Pellegrini-Masini et al. 2020).

For women, non-recognition injustices manifest in ongoing responsibilities and disproportionate impacts through collecting and cooking with low-cost or free sources of fuel (e.g. woodfuel) as a form of gendered energy poverty (Feenstra and Özerol 2021; Moniruzzaman and Day 2020). Misrecognition was evident in discussions on responsibility for alleviating injustices. Solutions to gender-based violence centred on female responsibility (and action) to prevent violence rather than solutions to tackle the complexities of legislation mandating protection of forested areas: a need to reduce deforestation, and ongoing (gendered) realities of fuelwood use in regions lacking electrification (Kojola and Pellow 2021).

### Procedural injustices

Inclusive decision-making about energy is vital to achieving fair electricity access. Our findings highlight a new focus on investment as a procedural justice issue, missing from similar exisitingresearch (Aung et al. 2021; Gamula et al. 2013; Kaunda 2013), our findings examine procedural injustices relating to investment. This reinforces other investment-based assessments of procedural justice (de Graeff et al. 2018; Thorpe 2018; Walker and Baxter 2017). Interviews revealed four barriers to procedural injustices in electricity access in Malawi: (i) a perceived financial risk by, and lack of financing from, foreign investors for energy; (ii) inefficient government schemes for energy investment and growth; (iii) inefficient management of utilities; and (iv) financing conditions to extending and maintaining services for the national grid.

Financial support from donors and foreign investors is critical for expanding capacity of, and access to, electricity from Malawi’s energy sector. Interviewees identified a lack of such financing as a significant procedural injustice (#1, #3, #4, #7, #8, #11, #12, #21), with descriptions of the energy sector as risky environment to invest in (#8, #20, #21). Several challenges compound perceptions of high risk; management and administrative staff face high turnover (#12, #19) with long-term changes harder to implement (#1). Furthermore, both a historical lack of economic growth (e.g. GDP) and limited industrial development impact on perceptions of financial risks associated with investing in Malawi (#2, #3, #5, #6, #7, #11, #12, #21). This is often translated into the assumption that ‘ability to pay is lacking’ and concerns over financial returns from investments in energy systems (#21). To overcome the procedural barriers to international financing, there is an urgent need to ‘de-risk investment’ (#8) in Malawi.

Secondly, perceptions of a lack of efficient and robust government policies for energy investment and growth are hampering fair electricity access in Malawi (#1, #3, #8, #7, #5, #11, #12, #20, #22). Energy policy and legislation was described as ‘(not) stable enough for investors for renewable energies’ (#22), with a ‘lack of a strong political will’ (#3) further entrenching a lacking coherent and robust regulatory environment for investment. The economic viability of energy projects is thwarted by conflicts between affordability of energy tariffs and pricing policies and a need for economic returns (#6): ‘for projects to be sustainable they need to be turned into workable business models’ (#11), and ‘current tariffs make it not viable to invest in solar’ (#15).

Regulatory frameworks hinder independent power producers and private sector energy solutions from developing (#20, #21, #22). Interviewee #1 described an environment where alternative energy sourcing solutions were prematurely dismissed by those involved in national energy planning, with the example of an energy inter-connector from neighbouring Mozambique highlighted by #1. The interviewees reinforced a lack of centralised planning and policy for electrification also a procedural injustice (#4, #6, #7, #8, #15, #16, #21), exemplified in what was described as a ‘piecemeal approach’ (#8) to energy system planning. The Malawian Rural Electrification Programme (MAREP) was also noted to have a ‘problematic’ (#21) approach to energy planning, with time lag in connection to grid (#15). MAREP has only connected eight out of 336 areas (Malawi 2018). Donor-driven mini-grids were described as interim solutions indicative of slow centralised planning (#5, #6, #17); ‘long-term solution is always to provide access to the grid’ (#6), and where sites selection for mini-grids is random (#22). Mini-grids themselves were cited as unsustainable approaches to rural electrification (#14, #16, #21). As one interviewee put it, it is ‘easy to build the grid but difficult to sustain itself’ (#19). This has led to a scenario in Malawi where development happens ‘upside down’ (#4), with utilities reactive rather than proactive at local level (#4, #16).

This mirrored what they described as occurring at a wider governance level. Inconsistency in policy, lack of implementation, and enforcement of policy were brought up throughout interviews (#5, #12, #16, #18); an interviewee elaborated further that ‘I believe […] policies should be talking the same language…(w)e have policies contradicting each other’ (#18). A lack of long-term sustainability and coherence of national energy policy were also identified as key concerns: ‘Malawi needs a sustained policy for at least a decade’ (#15) with questions remaining over ‘government policy [… and…] will it be consistent going forward?’ (#22). The inadequacy of planning and policy in centralising electrification efforts was symptomatic of a government with a ‘reputation for not following through with projects’ (#29). This lack of centralisation represents a procedural injustice, where access to electricity is random and future connection unsystematic.

Third, the inefficient management of national utilities ESCOM and EGENCO contributes to energy injustices. Despite an unbundling of ESCOM into two independent utilities, ESCOM (distribution) and EGENCO (generation), interviews revealed concerns over the historical and existing links between the two state managed entities (#3, #6, #14, #27). They described EGENCO as being unfairly preferred over other IPPs (#3) by ESCOM in plans to expand generation. Discussions highlighted a perception of inefficiency in the operation and management of both entities which slowed and prohibited access to electricity (#3, #6, #10, #12, #16, #20, #21, #30). With ESCOM constrained by lack of government financing and security of income (#2), delays in payments to EGENCO were common (#14). They described ESCOM as ‘one of the risky African utilities’ (#22) being ‘not financially solvent’ (#21). Together, the recent unbundling of ESCOM and the slow pace (2–3 years) of negotiating a power purchasing agreement (PPA) (#14, #20, #22) contributed to an environment of uncertainty for potential private sector investors: ‘still seeing how this unbundled entity (ESCOM) will work out’ (#22).

Finally, financing restrictions to extending and maintaining services for the domestic grid was a recurrent theme (#1, #2, #5, #11, #12, #19). Lack of security around financing hinders centralised planning (#2, #7, #12). ESCOM struggles to secure funding with little support from the government (#2) despite being ‘key’ to electrification aims (#7). Others were critical of government financing capacity (#3, #6, #25). One interviewee stated that ‘the government won’t manage to invest’ to meet electricity access targets of 30% by 2030 (#22). We move on to reflect on the key points raised in our analysis for making policy recommendations for Malawi.

## Conclusions and policy recommendations

**Fig. 3:**
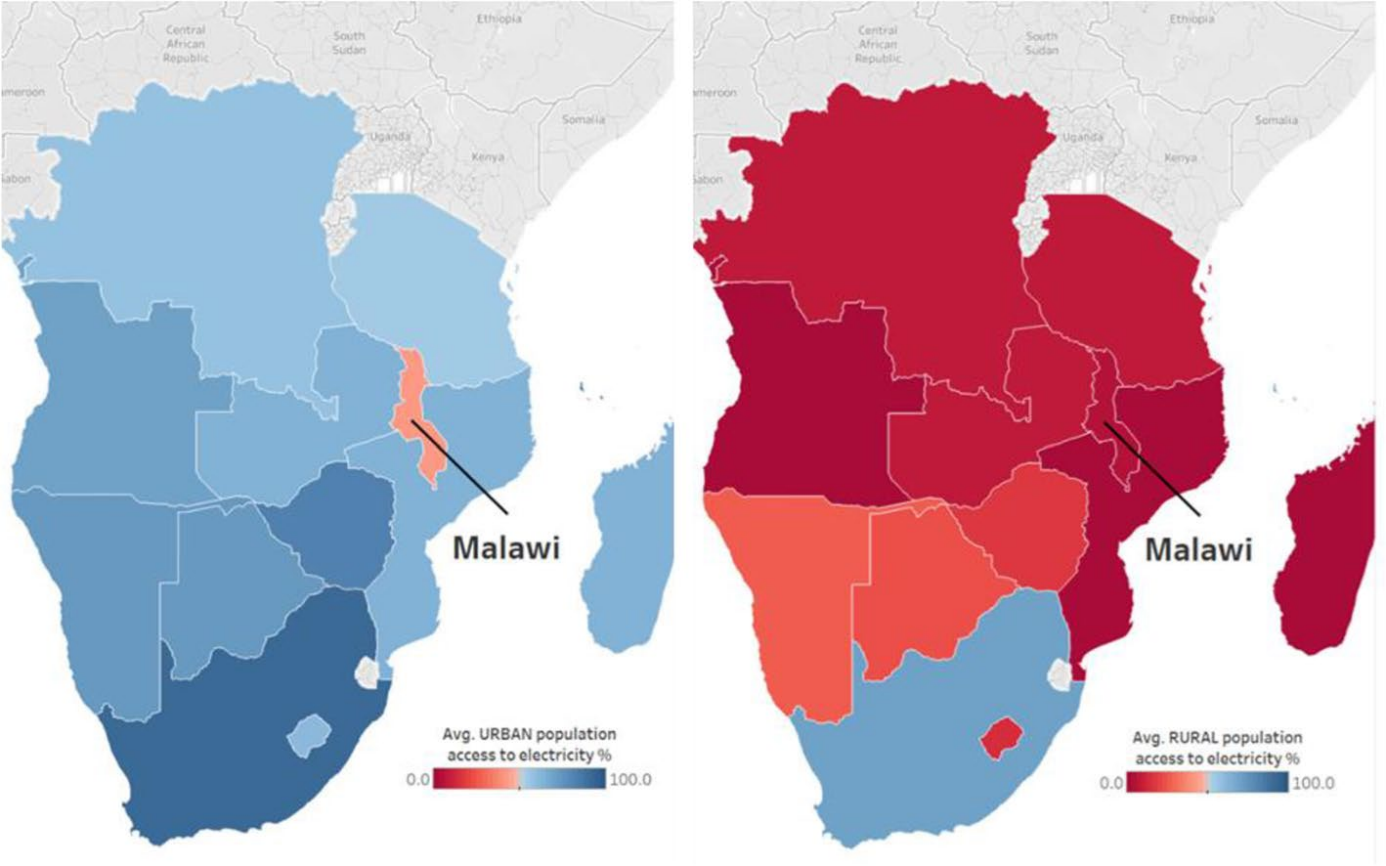
Urban and rural electricity access (%) in Malawi in the context of Southern Africa: This map shows United Nations Development Programme URBAN (left panel) and RURAL (right panel) population percentage access to electricity for 2010–2020 (UNSTATS 2021) as calculated by the authors. Population access to electricity is given as a percentage for each nation. UNDP data shows that Malawi is the only state in Southern Africa where less than half of the urban population has access to electricity. Poor rural electricity supply is widespread in the region, with South Africa the only state where most of its rural population has access to electricity

The focus of policymakers in Malawi is on increasing electricity access and access to clean cooking solutions throughout the country. However, there is a specific need in Malawi to address the low ability to pay for electricity in both rural and urban areas to stimulate demand and to support expansion in generation and transmission. There is widespread recognition of the different gendered impacts of ongoing fuelwood use in cooking, heating, and lighting (Smith et al. 2017), and policy must further seek to incorporate this into policies targeting deforestation and charcoal markets. But these policies need to focus explicitly on supporting greater rural access to electricity. Figure 3 below presents the stark contrast between rural and urban electricity access in Malawi and more generally the region.

### Reduce reliance on national government and utilities

Government and national private organisations in Malawi are both the drivers and victims of ineffective management. These actors adopt the position of barriers to change (Clayton et al. 2013; Walklate 2005; Wallimann-Helmer 2015). This is most apparent when we consider the role of national utilities. The division of distribution and generation was intended to open up electricity markets to new actors, to increase competition and lower costs. However, discussions indicated limited evidence for this, with repeated instances of inefficient operation and management of both sectors and the joint effect of maintaining the status quo. The increasing complexity of national utility management has resulted in further consolidation rather than expansion in the energy sector. This is a critical factor in slowing the adoption of renewable energy solutions and increasing electricity access.

### Attract new actors in decentralised off-grid energy development

One implication of this mismanagement has been the outsourcing of off-grid development. Outside organisations such as the World Bank and other international organisations drives in support of Trotter and Abdullah (2018), rural off-grid renewable energy policy in Malawi rather than national government. Many of these organisations, including the US Power Africa initiative, aim to increase rural electrification (Bos et al. 2018). The generation and connection targets, however, favour centralised electrification which benefits urban populations over rural populations. There is evidence of change however, with an increasing international push from donor agencies for off-grid electrification to meet the needs of rural and low-income populations (USAID 2019).

In Malawi, the breakup of utilities and the division of distribution and generation have crippled national energy policy making. This was leading to further mismatch between the national and local knowledge of project limitations and potential with the aims of international organisations. This reminds us that the ‘bullying’ of foreign sources in national energy management to promote renewables, as perceived in the literature (Monyei et al. 2018; Todd et al. 2019), can be rather symptomatic of an existing failed or doomed national management of the transition. The result is that we support calls for greater decentralisation (Lawrence 2020; Wiese 2020; Zalengera et al. 2020).

National utilities are, instead, hindering the development of new decentralised players on the market in off-grid renewables. The division of distribution and generation utilities and their management have not led to new community or local energy organisations emerging. National utilities and physical infrastructure continue to be reliant on fossil fuel industries and struggle to move away from this dependency (Mostert and van Niekerk 2018; Yenneti and Day 2016). In Malawi, at the national level, this manifested itself in an over-reliance on the proposed interconnector with Mozambique. This policy is a critical fall-back position where fossil fuel industries are well established in Mozambique. There was a continued interest in centralisation and electrification, rather than embracing renewable solutions beyond the development of solar parks in the North. We argue that private companies and governments, national or international, are strategic actors, but captured in a web of disorganisation, under-funding, and overall inertia. The low level of organisational capacity for managing large-scale energy transitions is beyond the existing actor networks in Malawi.

### Increase local energy democracy

In support of Cai and Aoyama (2018), we conclude that Malawi needs a more systematic policy towards encouraging community or local level renewable energy projects. This must include both a proper engagement with local governance structures and community owned investment. As raised by several interviewees, the current approach is too piecemeal, centrally controlled, and geographically dispersed. Investments in local renewable energy projects must, first, include a legal obligation of community ownership of both financial and technical resources and, second, engage in building local energy democracy. Existing research in Malawi presents a similar picture to that of our interviewees, namely, small-scale technology-based pilot schemes (such as lighting or cooking) as we find in Adkins et al. (2010) and Barry et al. (2011). In these schemes, the benefit is reduced to (often short-term) technological solutions with no sustained engagement with community governance structures. Renewable clean energy provision depends on comprehensive local empowerment.

### Improve local environmental management as a key driver for energy futures

The environment is central to determining energy futures in Malawi. Energy justice overlooks the importance of the local environment as a critical factor in policy development (McCauley et al. 2016). Our results underline the intimate connection between how the environment is managed and the effects of this on a society’s ability to visualise a different energy future. In Malawi, deforestation is not only a physical process. It is a collective national policy and community failure. Interviews reflected on the inability of policymakers and individuals to halt the devastating impacts of tree cutting. The male-dominated activity of turning trees into charcoal as a source of income drives an impossible vicious circle of income-based tree cutting (Owen et al. 2013; Smith et al. 2017; Zulu and Richardson 2013). The importance of empowering local environmental management and restoration is overlooked in policy solutions. The empowerment of local governance through fairer technological and financial investments must therefore be tied to protecting the environment and, for Malawi, reforestation.

This in-depth qualitative assessment of Malawi policymakers, representing a multi-sectoral, diverse group of commentators, has revealed the complexity involved in appreciating the interconnections between inequalities such as poverty, gender, social class with energy policymaking, physical systems, and infrastructures. Future research should try to engage other qualitative methods, and quantitative methods, in exploring the different dimensions of how grid systems interact with gendered realities. The success of a national policy affects future policy trajectories. Here, the failure in Malawi to cope with deforestation is hindering future energy developments. We need further research to explore such interconnections between energy policies and other sectors in Global South contexts to appreciate energy trajectories. Last, social inclusion is an integral but only one part of procedural injustices. We must understand inefficiency and incapacity to respond to challenges as important in procedural justice terms where an inability to act generates procedural inequalities. Research in this or other national contexts needs to explore the processes involved in such inequalities and their solutions.

The level of responsibility among low-income nations for avoiding or embracing fossil fuels is heavily contested. It is beyond the full consideration of this paper. We conclude with observations for the future of energy justice literature. The energy justice framework has allowed researchers to explore new empirical contexts like Malawi. Applying distribution, recognition, and procedural dimensions of energy justice offers an insightful framework for analysing such contexts. Energy justice is also normative, driving principles of good governance and new behaviours. Can the national energy policies and associated realities as outlined above ever be just if they ignore the intergenerational need to transition away from or resist adopting fossil fuels? Energy justice literature continues to overlook this critical question. The Malawian context as a pre-fossil fuel least developed nation is relevant for such consideration. We call for more ‘transition-aware’ work in such contexts.

## Appendix: List of organisations

African Trade Insurance Agency

Circle for Integrated Community Development (CICOD)

Community Energy Malawi

Cooperation Network for Renewable Energy in Malawi (CONREMA)

Department of Climate Change and Meteorological Services

Department of Energy Affairs

Department of Forestry

Electricity Generation Company Limited (EGENCO)

Energy Supply Corporation of Malawi Limited (ESCOM)

GIZ Malawi

Hara & Associates, Legal Practitioners

Malawi Congress of Trade Unions

Malawi Energy Regulatory Authority (MERA)

Malawi Environmental Endowment Trust (MEET)

Malawi Hydro Limited

Mount Mulanje Conservation Trust

Mulanje Electricity Generation Agency (MEGA)

National Association of Business Women (NABW)

NBS Bank

Renewable Energy Association of Malawi (REIAMA)

Rocky Mountain Institute

Sunny Money

UNDP Renewable Energy Partnership Group

UNICEF Renewable Energy

USAID

World Bank

## Footnotes

1 [2, 8, 12, 13, 15, 18, 21, 24, 26].

2 [4, 5, 7, 16, 17, 19, 20, 23, 29, 30].

3 [1, 3, 6, 9, 10, 11, 14, 22, 25, 27, 28].
